# Combination therapy of therapeutic antibody and vaccine or entecavir in HBV carrier mice

**DOI:** 10.3389/fmicb.2023.1173061

**Published:** 2023-05-05

**Authors:** Ruoyao Qi, Jiali Cao, Yangtao Wu, Xing Lei, Jinhang He, Liang Zhang, Rao Fu, Feng Chen, Yingbin Wang, Tianying Zhang, Ningshao Xia, Quan Yuan

**Affiliations:** ^1^State Key Laboratory of Molecular Vaccinology and Molecular Diagnostics, School of Life Sciences & School of Public Health, Xiamen University, Xiamen, Fujian, China; ^2^National Institute of Diagnostics and Vaccine Development in Infectious Diseases, Xiamen University, Xiamen, Fujian, China; ^3^Department of Clinical Laboratory, Women and Children’s Hospital, School of Medicine, Xiamen University, Xiamen, Fujian, China

**Keywords:** HBV, therapeutic antibodies, therapeutic vaccine, nucleotid analogs, combination (combined) therapy

## Abstract

Chronic infection with the hepatitis B virus (HBV) is a leading causes of liver cirrhosis and hepatocellular carcinoma. However, managing HBV treatments is challenging due to the lack of effective monotherapy. Here, we present two combination approaches, both of which aim to target and enhance the clearance of HBsAg and HBV-DNA. The first approach involves the use of antibodies to continuously suppress HBsAg, followed by the administration of a therapeutic vaccine in a sequential manner. This approach results in better therapeutic outcomes compared to the use of these treatments individually. The second approach involves combining antibodies with ETV, which effectively overcomes the limitations of ETV in suppressing HBsAg. Thus, the combination of therapeutic antibodies, therapeutic vaccines, and other existing drugs is a promising strategy for the development of novel strategies to treat hepatitis B.

## 1. Introduction

Since the discovery of the Australia antigen by Blumberg et al. in 1965, chronic hepatitis B (CHB) has been considered a neglectable public health concern for almost 60 years (Blumberg et al., 1965). However, the fact remains that 296 million people with CHB urgently require effective eradication therapeutics, as CHB is a leading cause of liver cirrhosis and hepatocellular carcinoma (Jeng et al., 2023). Currently, interferon-based therapies and nucleos(t)ide analogs (NAs) are the only two primary treatment options available for hepatitis B virus. Interferon-α and its pegylated version offered long-lasting virologic response with restricted effectiveness and frequent adverse effects (Ye and Chen, 2021). NAs are an effective treatment for reducing the levels of HBV-DNA in patients and are generally well tolerated. However, achieving functional cure of HBV, as indicated by HBsAg seroconversion, is rare achieved with NAs (Broquetas and Carrion, 2022). Collectively, development of effective therapeutics for CHB is still in urgent need.

As our understanding of chronic HBV infection deepens, it becomes clear that the primary obstacle to achieving a functional cure for HBV is the high level of peripheral HBsAg, which acts as a brilliant decoy to evade the immune system. During the past few decades, various monotherapies targeting HBsAg through distinct pathways have demonstrated promising but limited outcomes in both preclinical and clinical studies. Therapeutic vaccine, antibody and siRNA are the most commonly used types of monotherapies for treating CHB. In terms of HBV therapeutic vaccines, a minor decline of HBsAg has been observed in clinical trials of TG1050, BRII-179 and NASVAC (Zoulim et al., 2020; Akbar et al., 2021; Ma et al., 2021). Clinical trials of therapeutic antibodies such as HH-003, BRII-877, and VIR-3434 have been reported to transiently induce HBsAg loss by 1–2 log IU/mL. Similarly, comparable reduction of HBsAg has also been observed in clinical trials of siRNA such as JNJ-3989, arc-521, and VIR-2218 (van den Berg et al., 2020). While all the aforementioned monotherapies have shown inspiring outcomes, achieving the functional cure of HBV remains rare.

In addition to monotherapy, emerging combination strategies have demonstrated remarkable therapeutic efficacy. The effectiveness of therapeutic vaccines has been shown to increase through the knockdown of virus antigen expression *via* siRNA in preclinical studies (Michler et al., 2020). Moreover, effective inhibition of HBV expression has been reported through a combination of HBV targeted therapy and PD-1 immune checkpoint blockade (Zhen et al., 2021). Furthermore, treatment of HBV infection with nucleic acid polymers and peg-IFN has resulted in functional cure in 35% of participants (Bazinet et al., 2021). Thus, the strategic design of various monotherapy combinations may effectively attain functional cure for HBV.

In our preview studies, we reported several HBV therapeutic antibodies that target different domains of HBsAg. Among these mAbs, the E6F6 mAb binds to a linear epitope (aa 119–125 of HBsAg), while the 129G1 mAb recognizes the ‘second loop’ linear epitope (aa 137–151 of HBsAg; Zhang et al., 2016). Additionally, a bat HBV core antigen derived therapeutic vaccine presenting HBsAg-aa113-135(SEQ13) has been designated as a therapeutic vaccine (Zhang et al., 2020). Administration of all three of these interventions demonstrates substantial HBsAg and HBV-DNA loss in HBV-carrier mouse. Here, to assess the potential synergy effect of HBV therapeutic antibodies with other interventions, we evaluated the extent of HBsAg and HBV-DNA loss through the administration of mouse-originated and humanized HBV therapeutic antibodies in multiple mouse models.

## 2. Materials and methods

### 2.1. Mouse

For AAV-HBV mouse model, based on C57BL/6 strain, was developed using rAAV8-1.3HBV (ayw) purchased from Beijing FivePlus Molecular Medicine Institute Co. Ltd. Each mouse was intravenously injected with 2.5 × 10^10^ vg AAV-HBV. Assessments were conducted 4 weeks after AAV-HBV infection to establish immune tolerance. To facilitate the following assessments, a serum HBsAg titer ranging from 1 × 10^3^–2 × 10^4^ IU/mL was selected. To ensure that each group of AAV-HBV mice had the same baseline level of HBsAg, mice of the same age were divided into groups based on their serum HBsAg titer.

The HBV-transgenic (HBV-Tg) mouse, aged between 10 and 12 weeks, were kindly provided by Pei-Jer Chen (NTU, Taiwan; Wu et al., 2010). To enable the subsequent assessments, a serum HBsAg titer ranging from 1 × 10^3^–2 × 10^4^ IU/mL was selected. To ensure that each group of HBV-tg mice had a consistent baseline level of HBsAg, mice of the same age were divided into groups based on their serum HBsAg titer.

### 2.2. Antibodies

The 129G1 mAb were produced using hybridoma technology and characterized as previously described (Huang et al., 2012). Babl/c F1 Mice were subjected to intraperitoneal injections of paraffin oil to saturate peritoneal macrophages and prevent phagocytosis of antibody-secreting cells. After a 3-day incubation period, E6F6 hybridoma cells were administered intraperitoneally, and ascites were collected 3 to 7 days thereafter. The collected ascites was then subjected to centrifugation at 12,000 revolutions per minute for a duration of 10 min, followed by a 1:1 blending of the ascites supernatant with saturated ammonium sulfate. The mixture was allowed to precipitate on ice for a duration of 30 min before the precipitate was re-suspended in System A liquid of the Protein A column purification system. Subsequent centrifugation at 12,000 revolutions per minute for 10 min resulted in the discarding of the supernatant. The remaining solution was filtered and applied to a Protein A column for purification. The 162 and rc162 mAb used in this study were provided by our partner, Yangsheng TANG Co., Ltd.

### 2.3. Vaccines

The CRT3-SEQ13 gene fragment was synthesized by GENEWIZ, Inc. (Suzhou, China), and subsequently cloned into the pTO-T7 expression vector, which had been previously constructed in our lab (Luo et al., 2000). Following transfection of correct plasmids expressing CR-T3-SEQ13 into *Escherichia coli* strain ER2566 (Yang et al., 2005). The transfected ER2566 were cultured in an Erlenmeyer flask at 37°C for 4 h. Subsequently, IPTG was added, and the culture was incubated overnight at 16°C. The resulting precipitation was resuspended in lysis buffer containing 20 mM PB pH 6.0, 150 mM NaCl, and 5 mM EDTA. Sonication was performed for 3–4 min per bottle of bacteria, followed by centrifugation at 25,000 g for 20 min to collect the supernatant. The supernatant was heat-treated at 60°C for 20 min in a water bath, followed by another round of centrifugation at 25,000 g for 20 min to collect the supernatant. Saturated ammonium sulfate was slowly added to the supernatant in a 10:3 ratio (supernatant: ammonium sulfate) while stirring, and the mixture was left at 4°C for 2 h (or overnight). The mixture was then centrifuged, and the supernatant was discarded. The resulting precipitate was dissolved in PBS. If the precipitate did not dissolve after 10 min, DTT was added in a 5 mM concentration gradient up to a maximum of 20 mM. The solution was then filtered through a 0.22um filter and purified through ultracentrifugation using a sucrose density gradient (20, 40, 60, 80%) at 23,000 rpm for 10 h at 4°C. The desired protein was dialyzed in PBS, and the buffer was changed at least three times. The antigens were mixed with alum adjuvant using the protocol previously described (Zhang et al., 2015). The CR-T3-SEQ13 protein and vaccine formulation used in this study were kindly provided by our partner, Xiamen Innovax Biotech Co., Ltd.

To prepare the sample for electron microscopy, the protein was diluted in PBS at a 2x gradient. Negative staining of the protein solution was performed by placing a drop on a copper grid and staining with 1–2% phosphotungstic acid (PTA) at pH 6.5–7.0 for 5–10 s, followed by drying for observation under a transmission electron microscope.

### 2.4. Virological indicators

The HBV DNA levels in the mouse serum specimens were measured using a real-time qPCR assay from Premix Ex Taq™ (Takara, Dalian, China). The primer sequences were as follows: 5′-TTTCACCTCTGCCTAATCAT-3′ and 5′-TCAGAAGGCAAAA AAGAGAGTAACTC-3′. The probe sequence was 5′-HexCCTTGG GTGGCTTTGGGGCATGGA-1-3′.

The HBsAg chemiluminescent quantitation kit and HBeAg quantitation ELISA kit were obtained from Beijing Wantai Biological Pharmacy Enterprise Co., Ltd. The mouse serum was diluted at a ratio of 1:1000 using ED11. The chemiluminescence plate was equilibrated to room temperature, and 20 μL of the sample dilution was added. Following this, 100 μL of the sample and 5 standard samples (45, 9, 1.8, 0.36, and 0.072) were added and incubated at 37°C for 1 h. Subsequently, 50 μL of the enzyme-labeled secondary antibody was added and incubated at 37°C for 1 h. The plate was washed five times to remove any excess liquid, and any residual moisture was removed by shaking the plate dry. Finally, 100 μL of the color developing solution (A + B) was added, and the third reading was recorded. The actual IU value of the sample was calculated based on the standard samples.

The anti-SEQ13 and anti-CRT3-SEQ13 titers were detected using indirect ELISA kits developed by Beijing Wantai Biological Pharmacy Enterprise Co., Ltd. The anti-162 mAb titer was detected using indirect ELISA kits developed by Yangsheng TANG Co., Ltd. The recombinant HBsAg protein (CHO cell-derived, Wantai Biological Pharmacy Enterprise Co., Ltd., Beijing, China) or synthesized SEQ13 peptide (Jingju, Xiamen, China) was added at a concentration of 200 ng per well to coat the wells. Nonspecific binding was prevented by blocking with a solution of 2% bovine serum albumin (BSA) and 10% sucrose in phosphate-buffered saline (PBS). A series of sample dilutions ranging from 10 to 10,000 were prepared. During the assay, 100 μL of the specimens were added to the reaction well and incubated at 37°C for 60 min. Antibody titers was detected through horseradish peroxidase (HRP)-conjugated anti-mouse/human pAb (Thermo Scientific, Rockford, United States). Following the reaction of the chromogenic substrate and the stop solution, measure the absorbance of each well at 450 nm and 630 nm. The maximum dilution fold that yielded an OD450 nm- 630 nm value greater than 0.1 was used to calculate the antibody titer as follows: titer = (OD450 nm- 630 nm) /0.1 × dilution fold.

### 2.5. Quantification and statistical analysis

Statistical analyses were conducted using Prism 8 software (GraphPad). The bars rep-resent the mean. For datasets containing more than two groups, both normality and homogeneity of variance tests were performed. If these tests were passed, an ordinary one-way ANOVA with Tukey’s post-hoc test was performed to compare the means of each group. If only the normality test was passed, Brown-Forsythe and Welch ANOVA tests were used, followed by the Kruskal–Wallis test and Dunnett T3 test for post-hoc comparisons. If neither test was passed, the Kruskal–Wallis test and Dunn’s method were employed to compare the mean rank of each group. For datasets containing only two groups, an unpaired t-test was performed if both normality and homogeneity of variance tests were passed. If only the normality test was passed, an unpaired t-test with Welch’s correction was used. If neither test was passed, the Mann–Whitney test was performed to compare the mean rank of each group.

## 3. Results

### 3.1. Manufacturing and characterization of therapeutic agents for hepatitis B virus

The current investigation focuses on exploring the potential of the therapeutic vaccine CRT3-SEQ13 and therapeutic anti-HBV antibodies 129G1, E6F6, and 162 for managing hepatitis B virus (HBV) infection. These agents are promising candidates for treating chronic HBV infection, as they have exhibited significant inhibitory effects on HBV in various HBV-carrying mouse models and have induced high levels of anti-HBV humoral immune response (Zhang et al., 2016, 2020).

**Figure 1:**
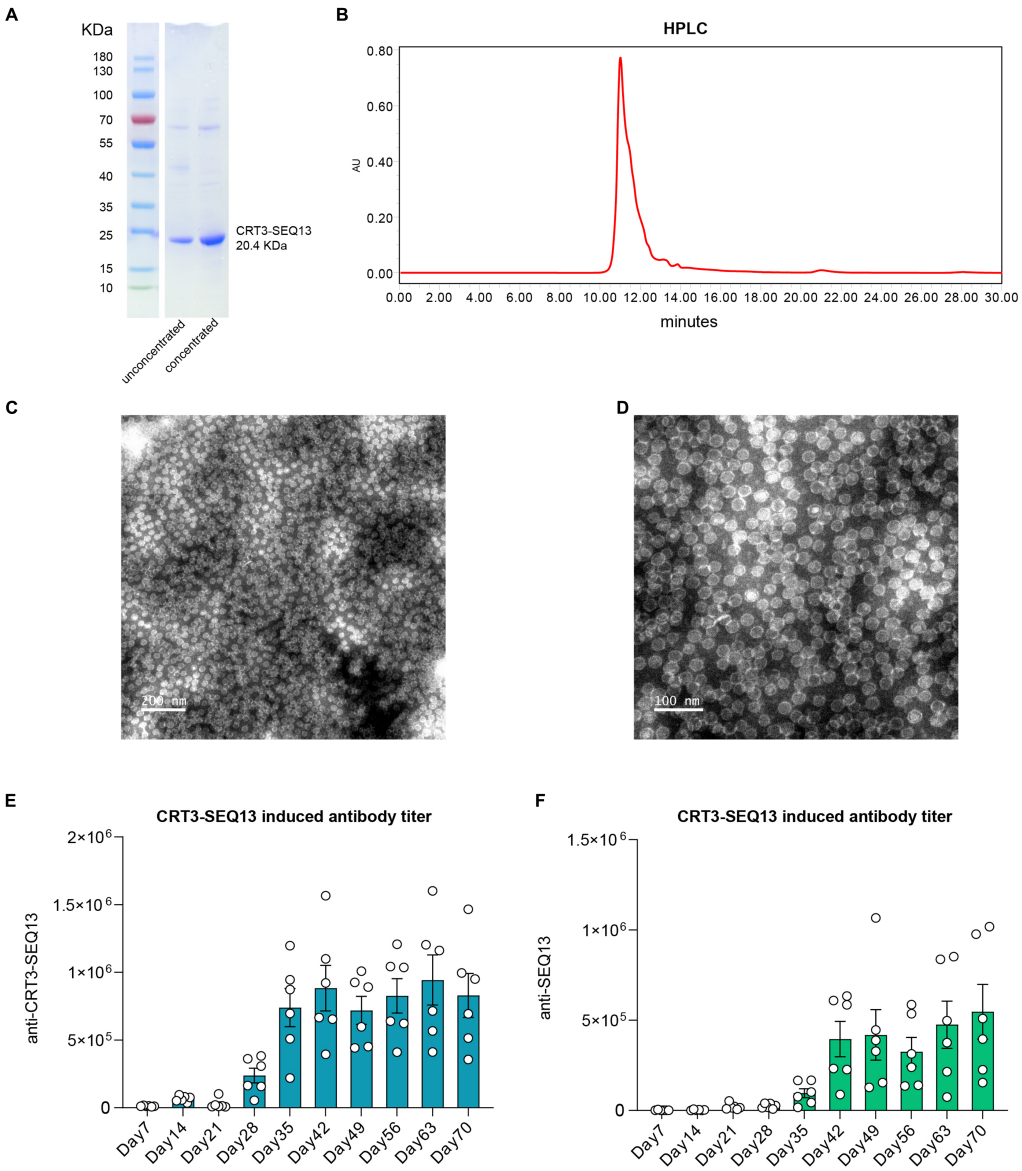
Characteristics of HBV therapeutic vaccine CRT3-SEQ13. **(A)** Western blot shown the purity and molecular weight of CRT3-SEQ13. **(B)** High performance liquid chromatography demonstrated the purity of CRT3-SEQ13. Electron microscope photo of CRT3-SEQ13 at **(C)** 200 nm and **(D)** 100 nm scale. 12 μg CRT3-SEQ13 was administrated at day 0, and day14 intramuscularly, **(E)** anti-CRT3-SEQ13, and **(F)** anti-SEQ13 titer were shown in wildtype mouse serum.

CRT3-SEQ13 exists as polymeric particles with 180 or 240 copies in PBS buffer. The protein purity of the four proteins was assessed using SDS-PAGE (Figure 1A), and their degree of polymerization was evaluated using HPLC (Figure 1B). The results showed that the protein purity was optimal, and the degree of polymerization met the expected standard. Since CRT3-SEQ13 is a virus-like particle (VLP), particle formation was assessed by transmission electron microscopy negative staining, and the results confirmed the expected particle formation (Figures 1C,D). To verify the immunogenicity of CRT3-SEQ13, wildtype C57BL/6 mice were administered intramuscularly. The serum anti-immunogen assessment demonstrated that two injections of CRT3-SEQ13 on day 0 and day 14 induced potent and sustained humoral response (Figures 1E,F). The HBV therapeutic antibodies, 129G1, and E6F6, were produced and characterized as reported (Zhang et al., 2016).

### 3.2. Administration of therapeutic antibodies over the long-term promotes functional cure

In our previous research on HBV therapeutic antibodies, we demonstrated that administration of antibodies can lead to prolonged suppression of HBV. However, it is unclear whether long-term antibody administration can promote functional cure of HBV.

**Figure 2:**
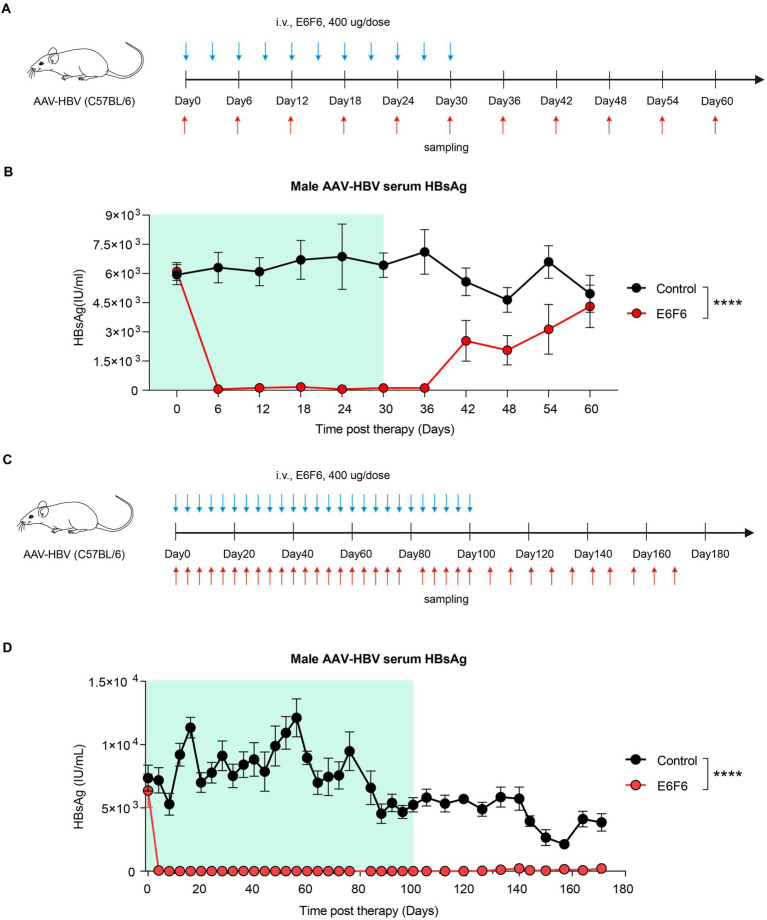
Sustained E6F6 administration eradicate HBV infection efficiently. **(A)** AAV-HBV mice (*n* = 8/group; HBsAg: 1 × 10^3^–2 × 10^4^ IU/mL) were administrated with E6F6 intravenously as indicated by blue arrows, the time of blood sampling was indicated by red arrows. Vehicle was injected intravenously as control. Schematic representation of the short-term E6F6 therapy procedure. **(B)** Time kinetic of HBsAg in male AAV-HBV mice during short-term E6F6 administration. **(C)** AAV-HBV mice (*n* = 4/group; HBsAg: 1 × 10^3^–2 × 10^4^ IU/mL) were administrated with E6F6 intravenously. Schematic representation of the long-term E6F6 therapy procedure. **(D)** Time kinetic of HBsAg in male AAV-HBV mice during long-term E6F6 administration. Results were representative of three independent experiments. Statistical analyses were performed between red line and black line. *p < 0.05; **p < 0.01; ***p < 0.001; ****p < 0.0001.

To address this question, we assessed short-term (Figure 2A) and long-term (Figure 2C) therapeutic antibody administration in an AAV-HBV mouse model. Short-term administration of E6F6 maintained mouse serum HBsAg at baseline levels during treatment, but HBsAg levels rebounded rapidly 12 days after the end of treatment and returned to baseline levels in 30 days (Figure 2B).

In contrast, long-term therapeutic antibody administration was highly effective, with sustained suppression of serum HBsAg over a monitoring period of 171 days (Figure 2D). The absence of rebound in serum HBsAg levels for 71 days after treatment suggests that these AAV-HBV mice may have achieved functional cure.

These results demonstrate that long-term administration of therapeutic antibodies can effectively eradicate HBV infection, while short-term administration is insufficient to achieve eradication. These findings are promising for the treatment of HBV in preclinical studies. However, frequent antibody injections may pose challenges for patient compliance during clinical practice. Therefore, multiple HBV combination interventions have been evaluated to facilitate HBV eradication and improve patient compliance.

### 3.3. Sustained suppression of HBsAg ameliorates therapeutic vaccine efficacy in central tolerance mouse model

**Figure 3:**
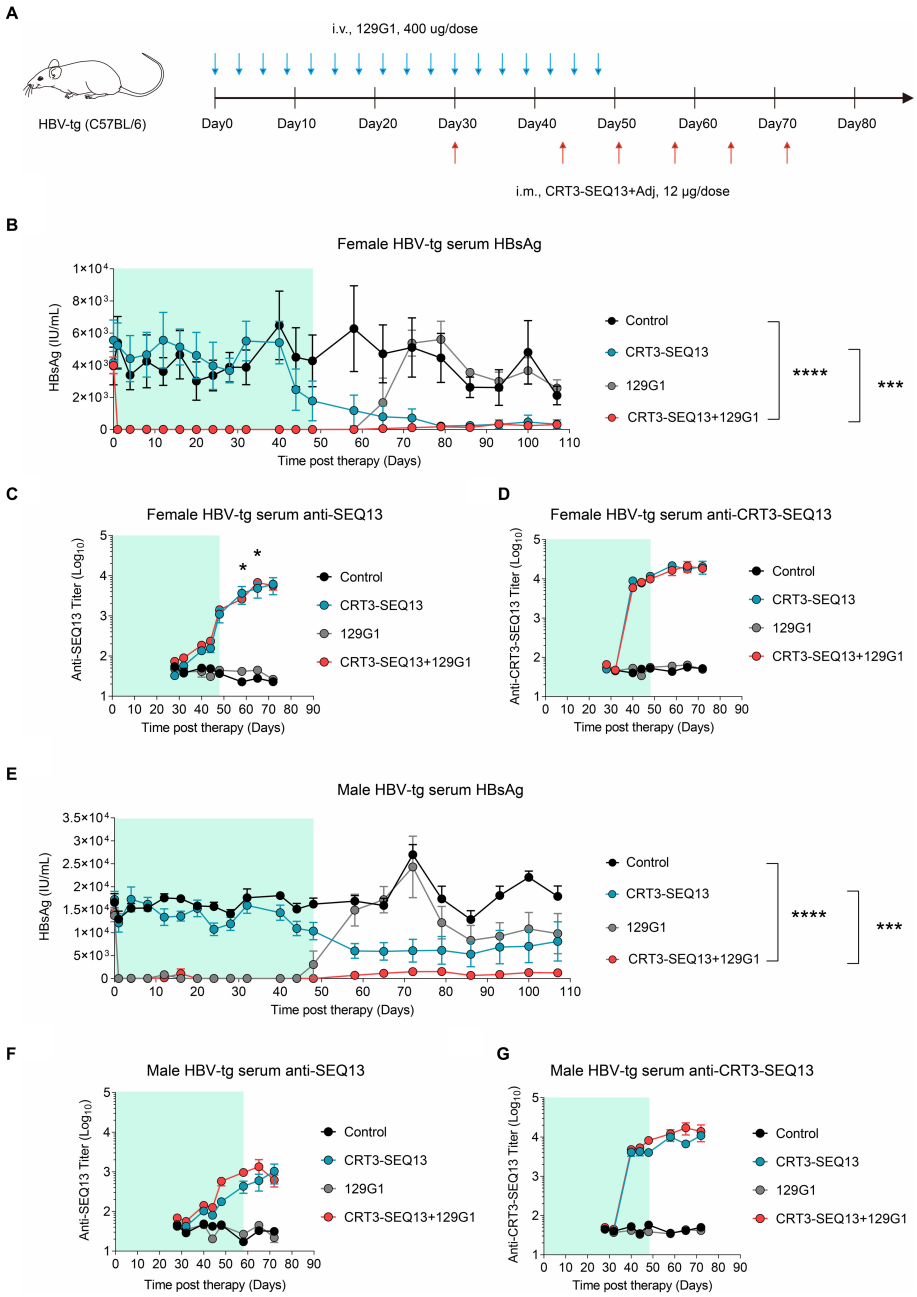
The combination of therapeutic antibody and vaccine breaks central immune tolerance in HBV-tg. **(A–G)** Central tolerance, HBV-transgenic mice (*n* = 4/group; HBsAg: 1 × 10^3^–2 × 10^4^ IU/mL) were administrated with 400 μg 129G1 intravenously every 3 days as indicated by blue arrows, 12 μg CRT3-SEQ13 was administrated at day 30, day 44, day 51, day 58, day 65, and day 72 intramuscularly as indicated by red arrows. Start of vaccination was day30. PBS was injected intravenously as control. **(A)** Schematic representation of the combination therapy procedure used for **(B–G)**. **(B–D)** Time kinetic of virological indicators in male HBV-tg mice. Serum levels of **(B)** HBsAg, **(C)** An-ti-SEQ13 antibodies, and **(D)** Anti-CRT3-SEQ13 antibodies. **(E–G)** Time kinetic of virological indicators in female HBV-tg mice. Serum levels of **(E)** HBsAg, **(F)** Anti-SEQ13 antibodies, and **(G)** Anti-CRT3-SEQ13 antibodies. Results were representative of three independent experiments. Statistical analyses were performed between red line and black line. *p < 0.05; **p < 0.01; ***p < 0.001; ****p < 0.0001.

In order to assess the potential synergy effect of HBV therapeutic vaccine and antibody, we conducted a sequential administration of CRT3-SEQ13 and 129G1 in HBV-transgenic (HBV-tg) mouse model. HBV-tg mice were carefully selected based on their serum HBsAg levels, ranging from 1 × 10^3^ IU/mL to 2 × 10^4^ IU/mL, which corresponds to the typical HBsAg titer observed in the majority of CHB patients. To ensure a thorough evaluation, the mice were subjected to a rigorous injection schedule: 17 injections of 129G1 monoclonal antibody at three-day intervals over a 48-day period, as depicted in Figure 3A, followed by six administrations of CRT3-SEQ13 on days 30, 44, 51, 58, 65, and 72. To account for the gender-related nature of HBV infection, we used both male and female HBV-tg mice to assess the therapeutic potential of this combination therapy.

Remarkably, in the female HBV-tg mouse, circulating HBsAg levels were maintained below 10 IU/mL following 129G1 administration in both the monotherapy and combination therapy groups (Figures 3B,E). As anticipated, serum HBsAg titers returned to baseline level after 129G1 was discontinued in 129G1 monotherapy group. It is noteworthy that the administration of CRT3-SEQ13 monotherapy resulted in a significant reduction of serum HBsAg levels from 5,500 IU/mL to 300 IU/mL in the female HBV-tg mouse model (Figure 3B). While both CRT3-SEQ13 monotherapy and combination therapy produced similar results in suppressing serum HBsAg at the endpoint of assessment, the combination therapy displayed superior efficacy in maintaining serum HBsAg levels throughout the 107-day study period. Moreover, there were no significant differences observed in epitope-specific (SEQ13) and vaccine-specific (CRT3-SEQ13) antibody levels between the CRT3-SEQ13 monotherapy group and combination therapy group in the female HBV-tg mouse (Figures 3C,D).

In male HBV-tg mouse, the administration of CRT3-SEQ13 monotherapy led to a significant decrease in serum HBsAg levels from 17,000 IU/mL to 6,000 IU/mL (Figure 3E). Furthermore, the combination therapy group achieved a much more substantial reduction in serum HBsAg levels, plummeting from 17,000 IU/mL to 1,200 IU/mL in the male HBV-tg mouse. This impressive decline was made possible by the efficient facilitation of HBsAg clearance through the administration of 129G1 mAb in combination therapy. Still, epitope-specific (SEQ13) and vaccine-specific (CRT3-SEQ13) antibodies did not show significant difference in CRT3-SEQ13 monotherapy group and combination therapy group in male HBV-tg mouse (Figures 3F,G).

These results indicate that HBV therapeutic antibody monotherapy is insufficient in sustaining the restraint of circulating HBsAg in a high HBsAg load scenario, but the administration of 129G1 monoclonal antibody over a period of 48 days significantly improves the efficacy of CRT3-SEQ13.

### 3.4. Prolonged repression of HBsAg promotes therapeutic vaccine efficacy in peripheral tolerance mouse model

**Figure 4:**
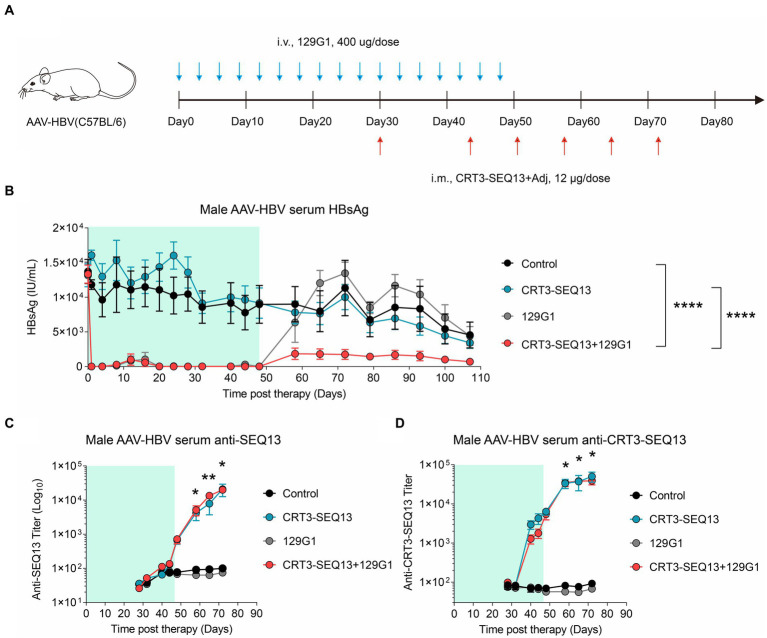
The combination of therapeutic antibody and vaccine facilitate serum HBsAg clearance in AAV-HBV **(A–D)** Peripheral tolerance, AAV-HBV mice (n = 5/group; HBsAg: 1 × 10^3^–2 × 10^4^ IU/mL) were administrated with 400 μg 129G1 intravenously every 3 days as indicated by blue arrows, 12 μg CRT3-SEQ13 was administrated intramuscularly at day 30, day 44, day 51, day 58, day 65, and day 72 as indicated by red arrows. Start of vaccination was day 30. PBS was injected intravenously as control. **(A)** Schematic representation of the combination therapy procedure used for **(B–D)**. **(B-D)** Time kinetic of virological indicators in AAV-HBV. Serum levels of **(B)** HBsAg, **(C)** anti-SEQ13 antibodies and **(D)** anti-CRT3-SEQ13 antibodies. Results were representative of three independent experiments. Statistical analyses were performed between red line and black line. *p < 0.05; **p < 0.01; ***p < 0.001; ****p < 0.0001.

In order to further validate the synergistic effect of HBV therapeutic vaccine and antibody, CRT3-SEQ13 and 129G1 were administrated sequentially in AAV-HBV mouse model (Figure 4A). The results showed that circulating HBsAg level in AAV-HBV was maintained below 10 IU/mL in both monotherapy and combination therapy groups upon 129G1 administration (Figure 4B). However, it was observed that serum HBsAg levels in the 129G1 monotherapy group quickly rebounded to baseline levels following the discontinuation of the 129G1 treatment on day 48. In contrast, the CRT3-SEQ13 monotherapy group did not display a significant decline in serum HBsAg titer in the AAV-HBV mouse model when compared to the control group. Interestingly, the combination therapy group exhibited a notable reduction in HBsAg levels, remaining under 2,000 IU/mL upon CRT3-SEQ13 administration, with two out of five mice exhibiting HBsAg levels under 100 IU/mL at the endpoint of assessment. Moreover, the immunogen-specific antibody titers in the AAV-HBV mouse model were found to rise rapidly within 7 days of CRT3-SEQ13 administration. Besides, both epitope-specific (SEQ13) and vaccine-specific (CRT3-SEQ13) antibody titers did not exhibit any significant differences between the CRT3-SEQ13 monotherapy group and combination therapy group (Figures 4C,D).

Collectively, these results demonstrate that the administration of 129G1 enhances the efficacy of CRT3-SEQ13 in the peripheral tolerance mouse model, showcasing the potential of this combination therapy in treating chronic hepatitis B infections.

### 3.5. Preclinical evaluation of humanized HBV therapeutic antibody

**Figure 5:**
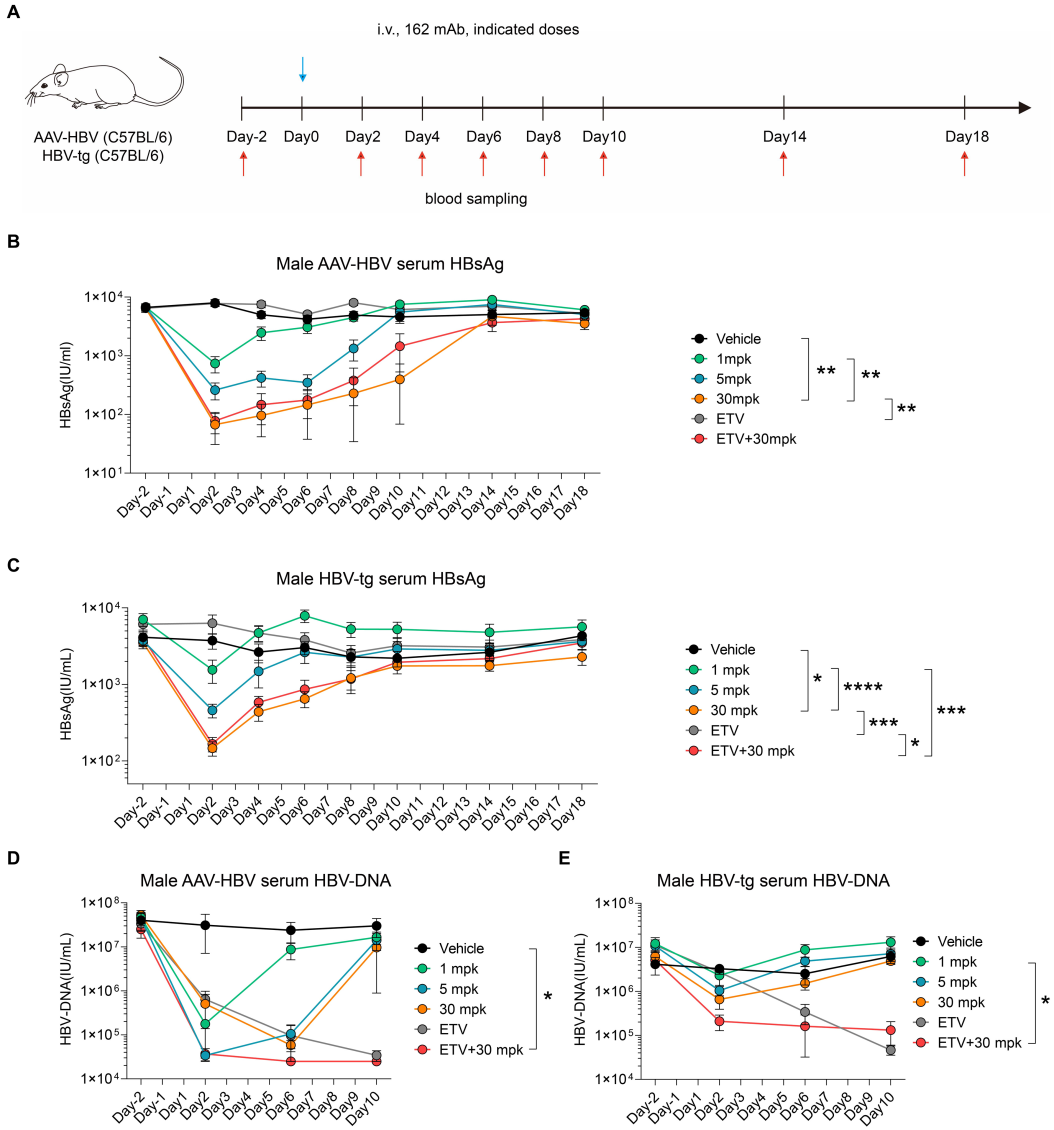
Humanized therapeutic antibody eradicate HBV in synergistic combination with ETV. **(A–E)** AAV-HBV and HBV-transgenic mice (*n* = 8/group; HBsAg: 1 × 10^3^–2 × 10^4^ IU/mL) were administrated with 162 intravenously as indicated by blue arrows, the time of blood sampling was indicated by red arrows. Vehicle was injected intravenously as control. **(A)** Schematic representation of the combination therapy procedure used for **(B–E)**. **(B,D)** Time kinetic of virological indicators in male AAV-HBV mice. Serum levels of **(B)** HBsAg, **(D)** HBV-DNA. **(C,E)** Time kinetic of virological indicators in male HBV-tg mice. Serum levels of **(C)** HBsAg, **(E)** HBV-DNA. Results were representative of three independent experiments. *p < 0.05; **p < 0.01; ***p < 0.001; ****p < 0.0001.

In order to fully explore the therapeutic potential of therapeutic antibody and entecavir (ETV), we conducted a series of preclinical experiments using both the AAV-HBV and HBV-tg mouse models. A single shot of humanized 162 mAb was administrated, experimental design is shown in Figure 5A. Specifically, different dosages of 162 mAb were administered, including 1 mg/kg (1mpk), 5 mg/kg (5mpk), and 30 mg/kg (30mpk), along with Entecavir (ETV) which was given *via* drinking to assess the synergy potential of 162 mAb and ETV. We also included a vehicle group which received the solvent of 162 mAb as control.

In the AAV-HBV mouse model, we observed a remarkable decrease in serum HBsAg levels from 6,600 IU/mL to 700 IU/mL, 300 IU/mL and 100 IU/mL in the 1mpk group, 5 mpk group and 30 mpk group, respectively, on day 2 post 162 mAb administration (Figure 5B). Similarly, in the HBV-tg mouse model, we observed a significant reduction in serum HBsAg levels from 4,100 IU/mL to 1,500 IU/mL, 500 IU/mL and 200 IU/mL in the 1mpk group, 5 mpk group and 30 mpk group, respectively, on day 2 post 162 mAb administration (Figure 5C). In both AAV-HBV and HBV-tg mouse model, ETV monotherapy did not affect HBsAg level, as expected.

We also assessed the HBV-DNA level of both the AAV-HBV and HBV-tg mouse models, as ETV mainly interferes with HBV reverse transcription. In the AAV-HBV mouse model, we observed a significant reduction in serum HBV-DNA levels from 4 × 10^7^ IU/mL to 1.8 × 10^5^ IU/mL, 3.4 × 10^4^ IU/mL, and 5.8 × 10^4^ IU/mL in the 1mpk group, 5 mpk group, and 30 mpk group, respectively, on day 2 post 162 mAb administration (Figure 5D). Similarly, in the HBV-tg mouse model, we observed a significant reduction in serum HBV-DNA levels from 4.1 × 10^6^ IU/mL to 2.3 × 10^6^ IU/mL, 1 × 10^6^ IU/mL and 6.6 × 10^5^ IU/mL in the 1mpk group, 5 mpk group and 30 mpk group, respectively, on day 2 post 162 mAb administration (Figure 5E). ETV monotherapy was effective in reducing serum HBV-DNA levels in both models, and the combination therapy of 162 mAb and ETV did not interfere with the therapeutic effect of each other.

Taken together, our findings suggest that the humanized 162 mAb demonstrated a promising therapeutic potential in both AAV-HBV and HBV-tg mouse models, and the combination therapy of 162 mAb and ETV might work in synergy to overcome the limitations of both monotherapies.

### 3.6. Maximum suppression of HBsAg achieved through reverse chimeric HBV therapeutic antibody administration weekly

**Figure 6:**
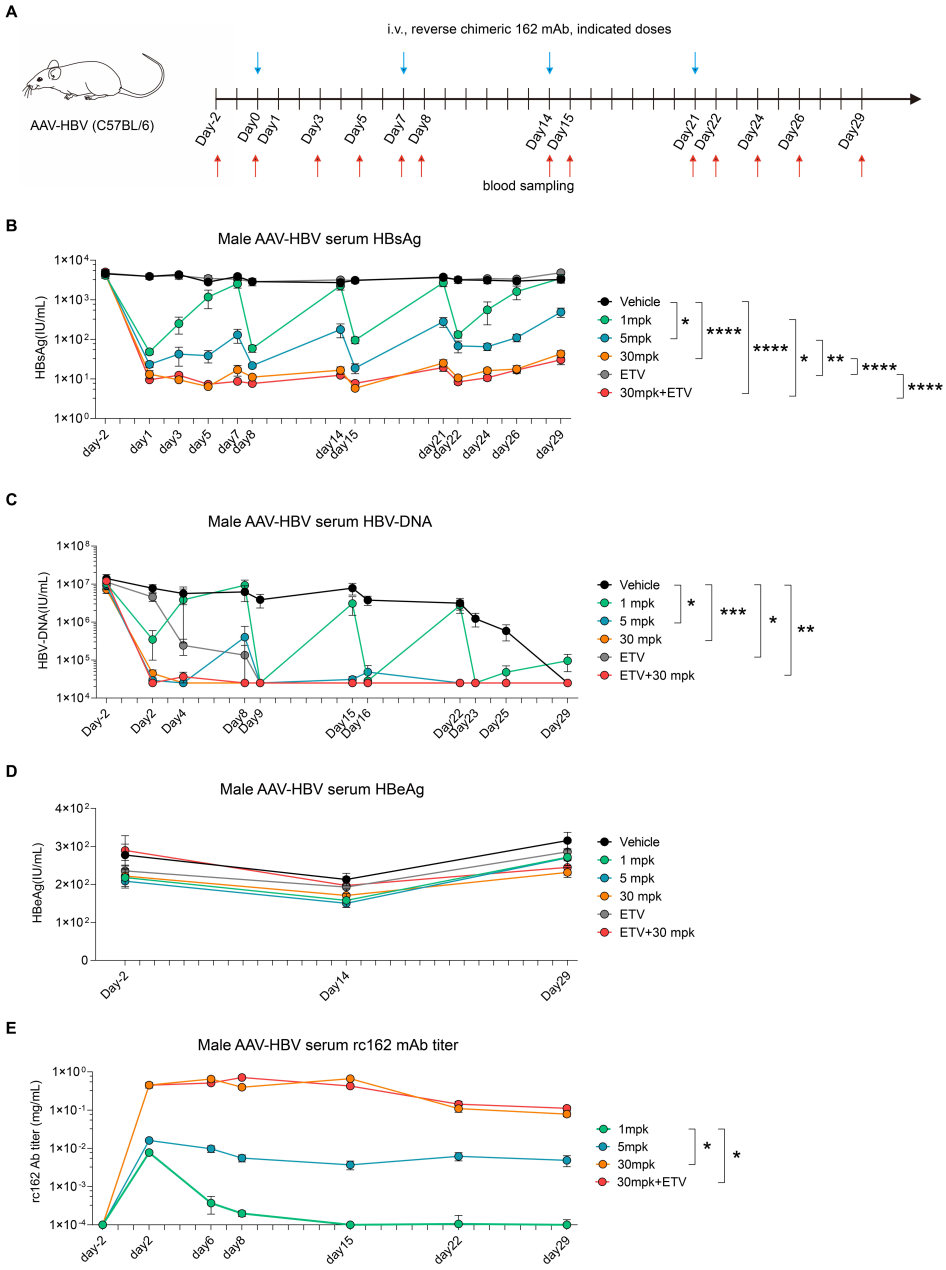
Sequential administration of reverse chimeric HBV therapeutic antibody suppresses HBsAg persistently **(A–E)** AAV-HBV mice (*n* = 8/group; HBsAg: 1 × 10^3^–2 × 10^4^ IU/mL) were administrated with rc162 mAb intravenously every 7 days as indicated by blue arrows, the time of blood sampling was indicated by red arrows. Vehicle was injected intravenously as control. **(A)** Schematic representation of the combination therapy procedure used for **(B–E)**. **(B, D)** Time kinetic of virological indicators in male AAV-HBV mice. Serum levels of **(B)** HBsAg, **(C)** HBV-DNA, **(D)** HBeAg, **(E)** 162 mAb. Results were representative of three independent experiments. *p < 0.05; **p < 0.01; ***p < 0.001; ****p < 0.0001.

To further investigate the pharmacodynamics (PD) and pharmacokinetics (PK) of 162 mAb in mouse, we designed a reverse chimeric 162(rc162) mAb, which was composed of mouse Fc and humanized Fab. We then administered four doses of rc162 mAb to AAV-HBV mouse models to assess its ability to retain serum HBsAg titer over time (Figure 6A). The results showed that serum HBsAg was significantly reduced from 4,500 IU/mL to 50 IU/mL, 20 IU/mL and 10 IU/mL in the 1mpk, 5 mpk, and 30 mpk groups, respectively, on day 2 post rc162 mAb administration (Figure 6B). Furthermore, the administration of rc162 mAb also led to a reduction in serum HBV-DNA levels from 1.4 × 10^7^ IU/mL to 3.5 × 10^5^ IU/mL, 3 × 10^4^ IU/mL, and 3 × 10^4^ IU/mL in the 1mpk, 5 mpk, and 30 mpk groups, respectively (Figure 6C). Besides, serum HBeAg levels did not show any significant difference after four doses of rc162 mAb administration over 29 days (Figure 6D). In terms of pharmacokinetics, the analysis of rc162 mAb showed a consistent antibody titer during the 29-day time course, which was almost the opposite trend of the HBsAg titer (Figure 6E).

All in all, these findings suggest that weekly administration of rc162 mAb can persistently suppress serum HBsAg and HBV-DNA, which makes the combination therapy of rc162 mAb and therapeutic vaccine a promising future prospect.

## 4. Discussion

Numerous preclinical and clinical trials for CHB indicated that monotherapy alone may be insufficient to achieve functional cure effectively. Previews studies have shown that several therapeutic antibodies against HBV results in significant decline in HBsAg levels upon administration. However, the suppression effect of HBsAg cannot be sustained as antibody administration stops. Therefore, it is crucial to reinvigorate host adaptive immunity to achieve functional cure effectively. Base on this theory, we have designed a combination strategy of HBV therapeutic antibody and vaccine to facilitate HBsAg clearance. The 129G1 mAb, which targets HBsAg, was administrated first to eliminate HBV-expressing cells and control circulating HBsAg. After 30 days of passive immunization, CRT3-SEQ13 was introduced to induce host active immunity. In male mouse model with high viremia and high antigenemia, our combination therapy demonstrated striking therapeutic efficacy compared to monotherapy. These promising results have given us the confidence to assess HBV therapeutic antibody in clinical trials. In order to do so, humanized and reverse chimeric forms of E6F6 mAb were generated and evaluated in HBV carrier mouse. Similar therapeutic effects were verified through pharmacodynamics and pharmacokinetics analysis. A randomized, double-blind, placebo-controlled phase I clinical study to evaluate the safety, tolerability, pharmacokinetics of 162 mAb with a single ascending dose in healthy adult subjects is currently active in Australia (NCT05310487). All in all, we demonstrated that prolonged administration of an HBsAg targeting antibody promotes therapeutic vaccine efficacy and is well-tolerated in human.

To assess the synergy effect of HBV therapeutic vaccine and antibody in the HBV carrier mouse, we evaluated the combination of CRT3-SEQ13 and 129G1 mAb due to the fact that E6F6 mAb directly binds CRT3-SEQ13. We assessed multiple combination strategies of E6F6 mAb and CRT3-SEQ13 in our pilot experiment. However, neither the administration of E6F6 mAb first in combination with CRT3-SEQ13 nor the administration of both E6F6 mAb and CRT3-SEQ13 at the same time showed a synergistic effect. Based on this observation, we selected the 129G1 mAb, which recognized HBsAg but did not bind to CRT3-SEQ13. In the second part, the humanized 162 mAb was constructed using the Fab of E6F6 mAb, as the E6F6 mAb demonstrated the most striking therapeutic effect among all candidates (Zhang et al., 2016). Moreover, the E6F6 mAb recognizes an evolutionarily conserved epitope (GPCK(R)TCT) of HBsAg and only forms a smaller immune complex. This unique binding characteristic of E6F6 mAb is important for HBsAg clearance through opsonophagocytosis *in vivo*.

In regard to the induced vaccine-specific antibody titer by CRT3-SEQ13, little difference was detected regardless of the administration of 129G1 mAb. These results may be perplexing as serum HBsAg titer did make a difference. Two possible explanations exist for this issue: First, the administration of the HBsAg therapeutic antibody is capable of eradicating HBV-expressing cells as we have previously demonstrated (Zhang et al., 2016). Second, we hypothesized that the quality of antibodies induced by CRT3-SEQ13 or the immune suppressive environment might be important for HBsAg clearance.

During the preclinical experiments of the humanized therapeutic antibody 162 mAb, only one shot was administrated due to host rejection. In our pilot experiments, significant anti-drug antibodies were developed when multiple shots of 162 mAb were administered to the subjects. The production of anti-drug antibodies might neutralize the therapeutic efficacy of 162 mAb and induce health problems in mice. To circumvent this issue, rc162 mAb consisting of a mouse Fc and humanized E6F6 Fab. Without the antigenicity from human Fc, rc162 can be administrated at multiple times without causing side effects. Furthermore, sustained HBsAg decline can be achieved through weekly administration of rc162 mAb.

As a sexually related disease, many studies suggest that males may exhibit certain symptoms of hepatitis B virus (HBV) infection more frequently than females. Specifically, males may exhibit higher HBsAg levels, more severe liver damage, cirrhosis, and a higher incidence of liver cancer resulting from chronic HBV infection when compared to females. This may be attributed to the fact that male hormones (such as testosterone) can contribute to liver damage. Sexual differences have also been observed in mouse models, with female mice displaying lower levels of HBsAg and HBV-DNA, making them more responsive to treatment. This explains why therapeutic vaccine administration alone is capable of eradicating HBV in female mice. Most of the assessments in this manuscript were conducted on male mouse models, which is a more stringent model, intended to emphasize the differences between the treatments.

In this study, we have evaluated the potential for synergistic effects of therapeutic antibody, therapeutic vaccine, and nucleoside analogs. In addition to the interventions mentioned above, several other HBV interventions could be considered as potential candidates in combination with E6F6 or CRT3-SEQ13. These include IFN-a (interferon-alpha), another drug marketed for the treatment of HBV, aside from NAs. Although the synergy effect of IFN-a and E6F6 or CRT3-SEQ13 has been assessed, little to no synergy effect has been observed. Another intervention is siRNA (small interfering RNA), which targets and degrades HBV mRNA. A combination strategy of siRNA administration followed by treatment with E6F6 or CRT3-SEQ13 demonstrated robust therapeutic efficacy. However, due to non-disclosure agreements, we are unable to present the data at this time. In summary, we believe that our therapeutic antibody and vaccine, in combination with other targeted therapies, may demonstrate similar synergy effects.

In the present study, we opted to conduct a 5-week monitoring period for assessing the therapeutic effect of E6F6 and CRT3-SEQ13 based on our pilot experiments. Previous studies have reported that the duration of therapeutic effect induced by these antibodies may range from 2 to 10 months (Zhang et al., 2020). However, in cases where the HBV infection is not completely eliminated, a rapid rebound of mouse serum HBsAg has been observed after the end of treatment. Considering the practical constraints of time and resources, we deemed it appropriate to limit the monitoring period to 5 weeks post-treatment. While we did not present data to support sustained loss of HBsAg for several months (3 to 6 months) after the end of treatment, the fact that CRT3-SEQ13 demonstrated sustained therapeutic effect for at least 40 weeks (Zhang et al., 2020) leads us to speculate that this combination therapy may show a similar sustained effect.

In conclusion, our data suggest that 162 mAb is an outstanding candidate for the treatment of CHB in combination with therapeutic antibody or nucleos(t)ide analogs. Our findings provide new insights into the design of a therapeutic strategy against persistent viral infection based on combination therapy of antibody and vaccine. With numerous promising HBV monotherapies currently under development, combining other anti-HBV candidates with 162 mAb may further facilitate the achievement of the global hepatitis elimination targets under the Sustainable Development Agenda 2030.

## Data availability statement

The original contributions presented in the study are included in the article/Supplementary material, further inquiries can be directed to the corresponding author.

## Ethics statement

The animal study was reviewed and approved by Ethics Committee of Xiamen University 32,170,943.

## Author contributions

RQ: conceptualization, investigation, and writing—original draft preparation. JC: methodology, data curation, and funding acquisition. YW (Yangtao Wu): resources and visualization. XL, LZ, and RF: validation. JH: software. FC: formal analysis. YW (Yingbin Wang): project administration. TZ: conceptualization, funding acquisition. NX: supervision and funding acquisition. QY: writing—review and editing. All authors contributed to the article and approved the submitted version.

## Funding

This research was funded by National Natural Science Foundation of China grants 32170943 (to TZ), 82102379 (to JC), and 31730029 (to NX); Natural Science Foundation of Fujian Province 2020J06007 (to TZ); Fujian provincial health technology project 2021QNB025 (to JC, suported by Xiamen Municipal Health Commission); and Xiamen Youth Innovation Fund Project 3502Z20206060 (to TZ).

## Conflict of interest

The authors declare that the research was conducted in the absence of any commercial or financial relationships that could be construed as a potential conflict of interest.

## Publisher’s note

All claims expressed in this article are solely those of the authors and do not necessarily represent those of their affiliated organizations, or those of the publisher, the editors and the reviewers. Any product that may be evaluated in this article, or claim that may be made by its manufacturer, is not guaranteed or endorsed by the publisher.

## Supplementary material

The Supplementary material for this article can be found online at: https://www.frontiersin.org/articles/10.3389/fmicb.2023.1173061/full#supplementary-material
